# Measuring PC activity in endocervical swab may provide a simple and non-invasive method to detect endometrial cancer in post-menopausal women

**DOI:** 10.18632/oncotarget.10287

**Published:** 2016-06-25

**Authors:** Sophea Heng, Andrew N. Stephens, Tom W. Jobling, Guiying Nie

**Affiliations:** ^1^ Implantation and Placental Development Laboratory, Centre for Reproductive Health, Hudson Institute of Medical Research, Clayton, Victoria, Australia; ^2^ Department of Molecular and Translational Sciences, Monash University, Clayton, Victoria, Australia; ^3^ Department of Biochemistry and Molecular Biology, Monash University, Clayton, Victoria, Australia; ^4^ Centre for Cancer Research, Hudson Institute of Medical Research, Clayton, Victoria, Australia; ^5^ Department of Obstetrics and Gynaecology, Monash University, Clayton, Victoria, Australia; ^6^ Epworth Research Institute, Epworth HealthCare, Richmond, Victoria, Australia

**Keywords:** endocervical swab, uterine lavage, PC activity, endometrial cancer, assay

## Abstract

Endometrial cancer is one of the most common gynecological malignancies in post-menopausal women. If detected at early stages, endometrial cancer can be effectively treated by abdominal hysterectomy. However, to date, there is no biochemical test available for early and easy detection of endometrial cancer. Our previous study has established that the total proprotein convertase (PC) activity is significantly increased in the uterine lavage of post-menopausal women with endometrial cancer. Uterine lavage can be obtained relatively non-invasively compared to uterine tissues, however, blood contamination and other factors limit the wide clinical use of uterine lavage. The aim of this study was to determine whether endocervical swab is a viable alternative to uterine lavage for the detection of endometrial cancer. We determined the correlation in PC activity between paired endocervical swabs and uterine lavages from individual post-menopausal women (control as well as endometrial cancer patients), and also compared the total PC activity in endocervical swabs between control and endometrial cancer patients. Our data demonstrated that the total PC activity in swab and lavage was highly correlative in post-menopausal women, and that the PC activity in endocervical swab was significantly increased in endometrial cancer patients compared to controls. These results strongly suggest that determining PC activity in endocervical swabs may provide a simple, non-invasive and novel method to detect endometrial cancer in post-menopausal women.

## INTRODUCTION

Endometrial cancer, the fourth most common malignancy [1], predominantly affects women in their post-menopausal years [2]. It is classified as type I or type II based on clinical, endocrine and epidemiological characteristics [3, 4]. Type I endometrial tumors are estrogen dependent, associated with endometrial hyperplasia and treatments lead to favorable outcomes [3]. Type II tumors are estrogen independent, associated with endometrial atrophy and have less favorable outcomes [3]. According to histopathological characteristics, the most common subtypes are endometrioid carcinoma, serous carcinoma, carcinosarcoma and clear-cell carcinoma [5].

Endometrial cancer can be treated effectively by abdominal hysterectomy, but early diagnosis is the key. Although a number of biomarkers such as chaperonin 10, pyruvate kinase M1, macrophage migratory inhibitory factor, and furin have been identified for endometrial cancer [6, 7], to date, no biochemical tests are available to diagnose endometrial cancer at early stages. Currently, endometrial cancer in post-menopausal women is often identified only when vaginal bleeding occurs, but by then the cancer is already well progressed. Thus a simple and non-invasive early detection method for endometrial cancer would fill a major gap in the management of endometrial cancer.

Malignant tumors are characterized by altered expression of a number of molecules that require post-translational activation by proprotein convertases (PCs) [8]. PCs are a family of serine proteases, seven of which (furin, PC1/3, PC2, PC4, PACE4, PC5/6 and PC7/8) share a consensus cleavage motif of (K/R-(X)n-(KR)↓(n = 2, 4 or 6, X is any other amino acid) [9]. PCs are ubiquitously expressed in normal conditions and have a number of functional roles in cancer [10]. We recently demonstrated that the well characterized PC furin is significantly up-regulated in the uterus of post-menopausal women with endometrial cancer [7]. Consistent with this, total PC activity was also significantly increased in uterine lavages of endometrial cancer patients compared to control post-menopausal women [7], raising the possibility of using uterine lavages to detect endometrial cancers [7]. However, even though uterine lavages can be collected relatively non-invasively [11–13], blood contamination and inconsistencies in sample recovery present practical barriers to the application of uterine lavage for routine clinical application in this context [14].

In this study, we aimed to establish a proof-of-concept that endometrial cancer can be detected by assaying the total PC activity in endocervical swabs as a viable alternative to uterine lavages. We first assessed whether the total PC activity in endocervical swab correlated with the uterine lavage in the same individual, and then evaluated the potential use of endocervical swabs for the detection of endometrial cancer.

## RESULTS AND DISCUSSION

### Correlation of PC activity in endocervical swabs and uterine lavages

**Figure 1:**
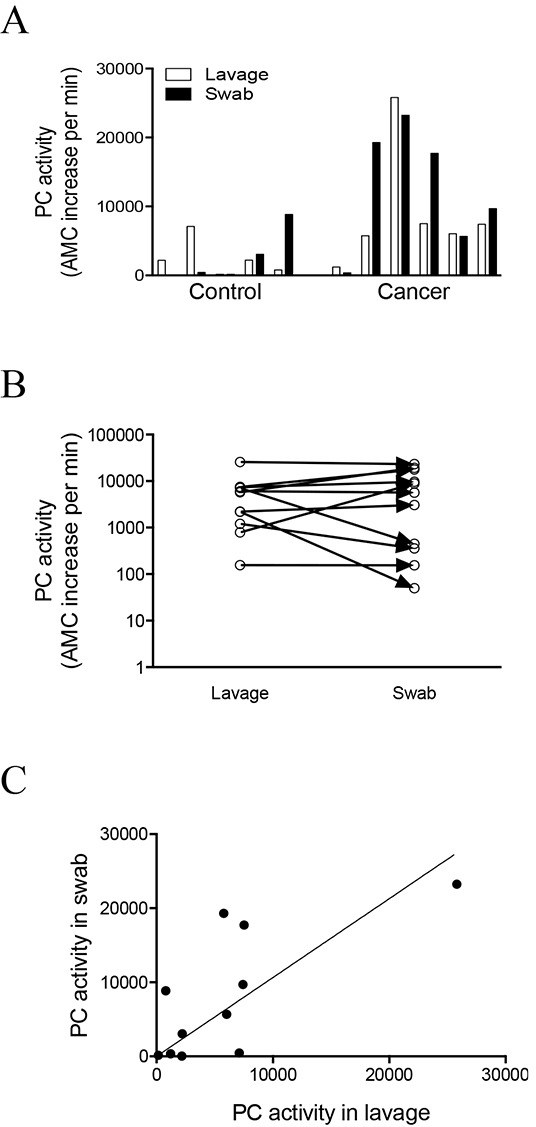
Correlation of PC activity between endocervical swabs and uterine lavages **A.** Total PC activity in the endocervical swab and uterine lavage pairs collected from post-menopausal control (n = 5) women and endometrial cancer (n = 6) patients. **B-C.** Correlation analysis between the swab and uterine lavage samples in all 11 pairs. (B) Pair-wise correlation analysis for each of the 11 swab and lavage pairs. (C) Global correlation between the swab and lavage samples, spearman r=0.68, *p*=0.025, 95% confidence interval 0.1185 to 0.9131.

We first explored whether the total PC activity in endocervical swabs is correlated with that observed in uterine lavages. Matched pairs of endocervical swab and uterine lavage samples were obtained from 11 post-menopausal women with (n = 6) or without (n = 5) endometrial cancer, and were assessed for total PC activity (Figure 1A). The pair-wise correlation of PC activity in these 11 swab/lavage pairs is shown in (Figure 1B). The global correlation analysis between swabs and lavages (n = 11) was significant (spearman r=0.68, *p*=0.025) (Figure 1C). This suggests that assaying total PC activity in endocervical swabs, which can be obtained much more simply and consistently than uterine lavages, may be a useful tool for the detection of endometrial cancer in post-menopausal women, as PC activity measured in the endocervical swabs reflected that measured in the uterine lavages. Importantly, PC activity was higher in both the swabs and lavages from women with endometrial cancer than controls (Figure 1A).

### Determining specificity of PC activity in endocervical swabs and uterine lavages

**Figure 2:**
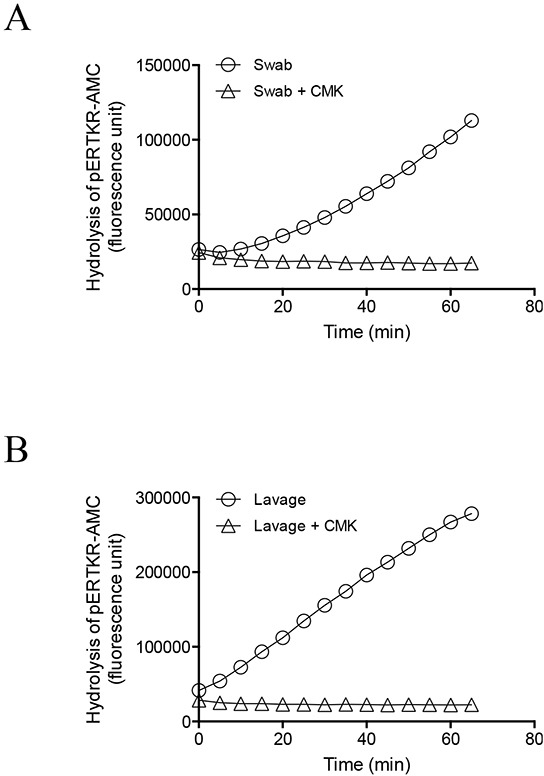
Inhibition of PC activity in endocervical swab and uterine lavage samples by a potent PC inhibitor, decanoyl–Arg-Val-Lys-Arg–chloromethylketone (CMK) Representative progressive curves of PC activity in the absence and presence of 100 μM CMK for swab **(A)** and lavage **(B)** are shown.

To confirm PC specificity, a representative endocervical swab and a uterine lavage from the same woman were analyzed for PC activity in the absence and presence of PC inhibitor dec-RVKR-CMK (CMK) (Figure 2). PC activity was completely abolished by CMK in both the endocervical swab (Figure 2A) and uterine lavage (Figure 2B), demonstrating PC specificity.

### Analysis of PC activity in endocervical swabs to detect endometrial cancer

**Figure 3:**
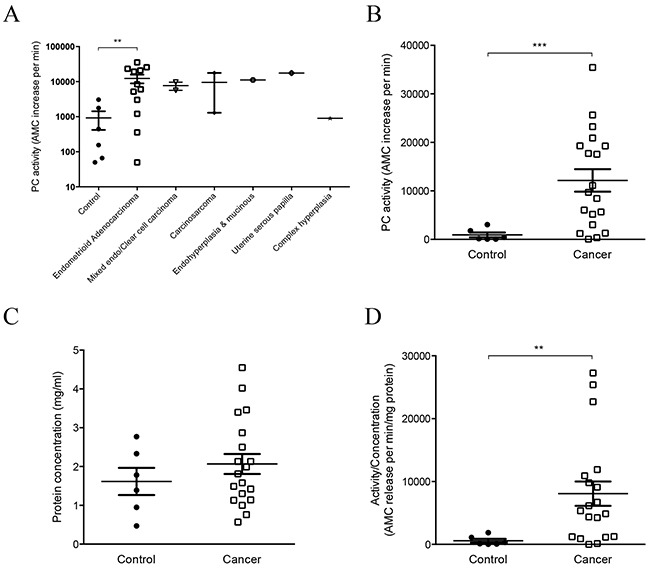
Analysis of PC activity in endocervical swabs collected from post-menopausal control women and patients with different types of endometrial cancer **A.** PC activity in control (n = 6), endometrioid adenocarcinoma (n = 12) and other types of endometrial cancer (n = 1-2 per type) patients. PC activity was significantly higher in endometrioid adenocarcinoma compared to controls, **p=0.0067. Data are expressed in log scales. **B.** Comparison of PC activity between control (n = 6) and all endometrial cancer analyzed as one group (n = 19). PC activity in endocervical swabs was significantly higher in cancers than controls, ***p=0.0001. **C.** Comparison of protein concentration between the two groups shown in B. **D.** The PC activity normalized to total protein concentration was also significantly higher in cancers than controls, **p=0.004.

We then determined total PC activity in a total of 25 endocervical swabs collected from control post-menopausal women (n = 6) and women with endometrial cancer (n = 19). Women with endometrioid adenocarcinoma (n = 12) showed significantly higher PC activity than controls (n = 6) (Figure 3A). Other types of endometrial cancer, such as mixed/clear cell carcinoma, carcinosarcoma, endohyperplasia and mucinous, uterine serous papilla and complex hyperplasia, all showed higher PC activities than controls. However, small sample size prevented statistical analysis of each of these cancer types (Figure 3A); our limited analysis showed no significant difference between type 1 and type 2 endometrial cancer. Future studies with larger sample sizes will further investigate this. When all types of endometrial cancer were analyzed as one group (n = 19), PC activity was significantly elevated in cancer (Figure 3B).

We also determined the concentration of total proteins in each swab, as each sample contains thousands of other proteins in addition to PC proteins. No significant differences in total protein concentration were detected between the controls and cancers (Figure 3C), although a few cancer swabs had higher protein concentration (Figure 3C). When the PC activity was normalized to the total protein concentration for each sample, the cancer group was still significantly higher than controls (Figure 3D), and the pattern was not altered by this normalization.

**Figure 4:**
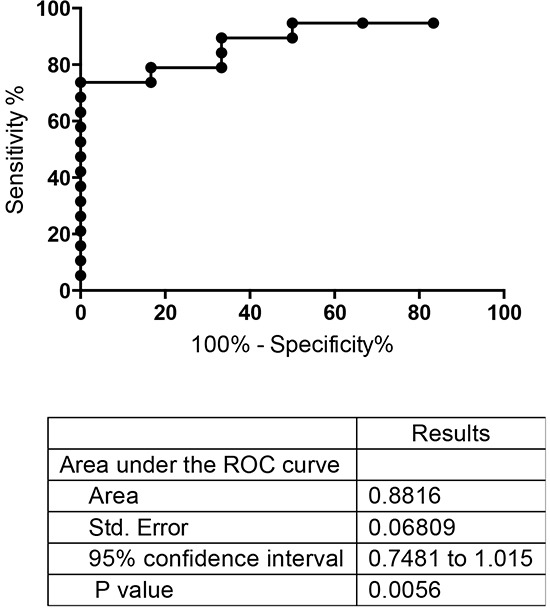
ROC curve analysis of total PC activity in endocervical swabs collected from post-menopausal control and endometrial cancer women Swabs from controls (n = 6) and endometrial cancers (all types, n = 19) shown in Figure 3B were analyzed.

The potential utility of measuring PC activity in endocervical swabs to detect endometrial cancer was then determined by a receiver operating characteristic (ROC) analysis (Figure 4). The area under the ROC curve was 0.88 (95% confidence interval of 0.75-1.01, *p*=0.0056). At a cut-off value of >4125, the sensitivity was 74% and specificity was 100%. When the cut-off value was reduced to >2400, the sensitivity was 80% and specificity was 83%. These results demonstrate that determining PC activity in endocervical swabs may provide a clinically useful yet very simple screening tool to diagnose endometrial cancer in post-menopausal women.

Our previous study has reported that detecting PC activity in uterine lavage can indicate the presence of endometrial cancer in post-menopausal women [7]. This current study extended our previous observation, and demonstrated that endocervical swabs provided similar efficacy for detecting endometrial cancer, without the associated drawbacks of the lavage sampling technique. For each individual woman, the PC activity in the endocervical swab was highly correlative to the uterine lavage. Importantly, PC activity in endocervical swabs is significantly higher in endometrial cancer patients than controls. Furthermore, the PC activity can be assayed in 96-well plates without the involvement of any specific antibodies. We therefore propose that assaying PC activity in endocervical swab may provide a novel, non-invasive and a high throughput screening method for the detection of endometrial cancer in post-menopausal women.

In summary, this study demonstrates that the total PC activity in endocervical swab and uterine lavage is highly correlative, and that the overall PC activity in endocervical swab is significantly elevated in endometrial cancer patients than controls. Monitoring PC activity in endocervical swab may thus provide a simple and novel tool for screening endometrial cancer.

## MATERIALS AND METHODS

### Collection of endocervical swabs and uterine lavages

Sample collection was approved by appropriate Institutional Ethics Committees (MMCB02031B and MMC06032C) and written informed consent was obtained from individual patients. The endocervical swab was collected by placing a cotton-based swab into the endocervical neck for 1 minute to absorb the fluid surrounding that area, including uterine fluid trickled down from the uterine cavity. Each swab was then suspended in 500 μl of saline and filtered through a 0.2 μm filter by centrifugation at 4°C for 10-30 min at 4000 g. The flow through was collected, aliquoted and stored at −80°C. Immediately after the swab collection, uterine lavage was obtained from the same person as previously described [7]. In brief, 5 ml of saline was infused transcervically into the uterine cavity with a fine catheter. The fluid was aspirated, centrifuged at 1000 g for 5 min to remove cellular debris, aliquoted and stored at −80°C. Endocervical swabs and uterine lavages were collected from post-menopausal control women (n = 6) and women with endometrial cancer (total n = 19; endometrioid adenocarcinoma, n = 12, mixed endometrial/clear cell carcinoma and carcinosarcoma, n = 2, and n = 1 for endohyperplasia and mucinous, uterine serous papilla and complex hyperplasia).

### Analysis of PC activity and protein concentration in endocervical swabs and uterine lavages

Total PC activity in endocervical swab and uterine lavage samples was determined by cleavage of a fluorogenic peptide substrate pERTKR-AMC (Bachem, Torrance, CA, USA) [15]. Neat samples (50 μl) were incubated at 37°C with 100 μM (final concentration) substrate in ½ area 96-well plates. The real-time kinetics of substrate hydrolysis, resulting in the release of AMC, was monitored every 5 min at excitation/emission of 355/460 nm (Wallac Victor 2 spectrophotometer, PerkinElmer, MA, USA); the rate of substrate hydrolysis (AMC released per min) was then calculated from the linear phase of each real-time kinetics progression curve. In a separate experiment to confirm assay specificity, PC activity in representative swabs and lavages was assayed in the absence and presence of the PC inhibitor decanoyl–Arg-Val-Lys-Arg–chloromethylketone (dec-RVKR-CMK, otherwise referred to as CMK) (Bachem, Bubendor, Switzerland) [7]. Total protein concentration of each swab and lavage sample was determined by nanodrop (ND-1000, Biolab group, Australia).

### Statistical analysis

Data were expressed as mean ± SEM and normality was determined using the D'Agostino & Pearson omnibus normality test. Statistical analysis used unpaired Student t-test (PRISM, version 6.00, GraphPad software, San Diego, CA). Receiver Operator Characteristic (ROC) curve determined the potential use of PC activity in endocervical swabs to distinguish endometrial cancer patients from the controls. **p<0.01 was taken as significant and ***p< 0.0001 was considered highly significant.
